# Next Generation Inactivated Polio Vaccine Manufacturing to Support Post Polio-Eradication Biosafety Goals

**DOI:** 10.1371/journal.pone.0083374

**Published:** 2013-12-12

**Authors:** Yvonne E. Thomassen, Aart G. van ’t Oever, Monique G. C. T. van Oijen, René H. Wijffels, Leo A. van der Pol, Wilfried A. M. Bakker

**Affiliations:** 1 Institute for Translational Vaccinology (Intravacc), Bilthoven, The Netherlands; 2 Bioprocess Engineering, Wageningen University, Wageningen, The Netherlands; Instituto Butantan, Brazil

## Abstract

Worldwide efforts to eradicate polio caused a tipping point in polio vaccination strategies. A switch from the oral polio vaccine, which can cause circulating and virulent vaccine derived polioviruses, to inactivated polio vaccines (IPV) is scheduled. Moreover, a manufacturing process, using attenuated virus strains instead of wild-type polioviruses, is demanded to enhance worldwide production of IPV, especially in low- and middle income countries. Therefore, development of an IPV from attenuated (Sabin) poliovirus strains (sIPV) was pursued. Starting from the current IPV production process based on wild type Salk strains, adaptations, such as lower virus cultivation temperature, were implemented. sIPV was produced at industrial scale followed by formulation of both plain and aluminium adjuvanted sIPV. The final products met the quality criteria, were immunogenic in rats, showed no toxicity in rabbits and could be released for testing in the clinic. Concluding, sIPV was developed to manufacturing scale. The technology can be transferred worldwide to support post polio-eradication biosafety goals.

## Introduction

Vaccines that provide protection against poliomyelitis have been available for decades [1]. Yet large efforts are undertaken in WHO’s global polio eradication initiative (GPEI) to obtain the next generation vaccines that are safe and available at low costs [2]. These vaccines are needed both for the ‘endgame’ in polio eradication and after eradication to prevent the risk of a global outbreak due to accidental or deliberate re-introduction of the virus. One of the anticipated next generation vaccines is an inactivated polio vaccine (IPV) based on the attenuated Sabin poliovirus strains resulting in a so-called Sabin-IPV (sIPV) [3]. The Sabin polioviruses (PV) are currently used in live oral polio vaccines (OPV) [4] and will provide additional bio-safety, over the wild-type viruses, during the manufacturing process [5]. Bio-safety requirements are becoming more stringent as new containment guidelines are drafted by the WHO’s Global Action Plan for Wild Poliovirus Laboratory Containment III (GAPIII) [6]. Future production and quality control of IPV using wild-type strains will require at least biosafety level 3 facilities [7]. This will not only increase manufacturing costs but will also limit the possibility of IPV manufacturing in low- and middle income countries for instance due to requirements on immunization coverage. The use of alternative strains like Sabin PV would require less stringent biocontainment, is encouraged by the WHO [5] and allows manufacturing in low- and middle income countries, which potentially lowers manufacturing costs [8]. Moreover, the use of an IPV instead of OPV will prevent the emergence of circulating vaccine-derived PV (cVDPVs), which may potentially re-seed the world with PV and negate the GPEI accomplishments [9]. 

**Figure 1:**
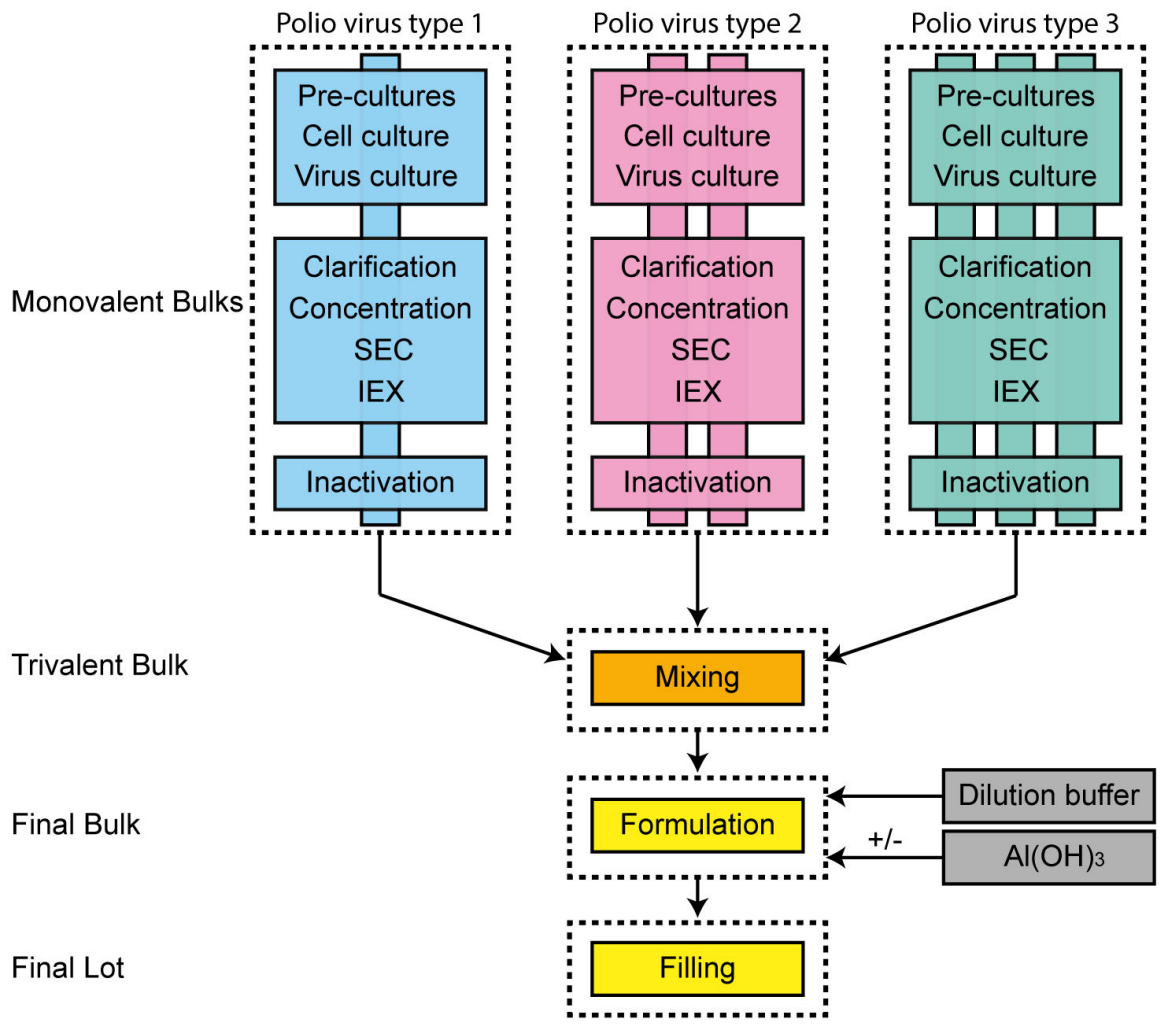
Process overview for preparation of trivalent IPV. Monovalent bulks are prepared for each PV (type 1, 2 and 3) separately. During monovalent bulk preparation Vero cells are expanded using two pre-culture steps and a cell culture followed by virus culture. Virus is purified using normal flow filtration for clarification, tangential flow filtration for concentration and two chromatography units, size exclusion and ion exchange chromatography. Purified virus is subsequently inactivated using formaldehyde. Subsequently these are mixed to obtain trivalent bulk prior to formulation and filling.

The development of the currently used IPV production process (for a process overview see Figure 1) dates back to the 1960s when at the RIV in Bilthoven a process was developed based on micro-carrier technology and primary monkey kidney cells [10,11]. This process was scaled-up to 350-L and later 750-L bioreactors. Additionally, the Vero cell line was introduced to replace the then used tertiary monkey kidney cells. To support manufacturing and increase the knowledge on IPV manufacturing, efforts like multivariate data analysis and the development of scale-down models, i.e. lab-scale equivalents of the manufacturing-scale processes, have been undertaken [12,13]. The availability of scale-down models, unique in the vaccine world, allows rapid assessment of process changes. 

Based on our vast history in IPV development and production [11], our previous experience with sIPV [14] and technology transfer [15-17] a project for the development and technology transfer of sIPV manufacturing under supervision of the WHO was initiated. Initially a proof-of-principle study was performed. In this study, sIPV was prepared from OPV as virus source. The three PV sub-types, obtained separately (Bio Farma, Indonesia), were concentrated, purified and inactivated and shown to yield a sIPV that was immunogenic in an animal model [3]. In principle, OPV manufacturers could, by acquiring correct downstream processing (DSP) equipment, produce sIPV. However, larger quantities of virus harvest (100-800 fold of current production quantities) are needed and upstream processing (USP) should be scaled-up [18].

Here we report the results of limited (to be able to quickly show proof of concept) process development for sIPV based on the established IPV production process, the subsequent manufacturing of clinical lots, their stability and pre-clinical studies. This work resulted in a vaccine that has recently been tested in the clinic (phase I/IIa) [19,20]. 

## Methods

### Ethics statements regarding animal studies

The abnormal toxicity study in suckling mice and guinea pigs and immunogenicity tests in rats used in this study were agreed upon by the Committee on Animal Experimentation of the Netherlands Vaccine Institute (Bilthoven, the Netherlands) (Study Permit numbers AAP 201000262, 201000302, 201000303, 2010000304, 2010000305, 2010000306, 2010000307, 201000310, 201100030, 201100054, 201100056, 201100101, 201100151, 201100170, 201100195, 201100214, 201100289, 201100345, 201200137, 201200154, 201200227 and 201200262). Animal handling in this study was carried out in accordance with relevant Dutch national legislation, including the 1997 Dutch Act on Animal Experimentation.

The protocol for the toxicity study in rabbits was reviewed and approved by the Animal Welfare Officer and Ethical Committee of WIL Research Europe B.V. (former name: NOTOX B.V.) as required by the Dutch Act on Animal Experimentation (Study Permit Numbers: DEC 08-48 and 10-18). The OECD guidance document on humane endpoints (ENV/JM/MONO/ 2000/7) is applicable for all animal studies carried out at WIL Research Europe B.V.. No distress or discomfort was noted during this study.

### Lab scale experiments

#### Upstream processing

Vero cells obtained from WHO (10-87) originally derived from ATCC (CCL-81) were used as host for PV production. Sabin PV type 1 (LSc 2ab KP_2_), Sabin PV type 2 (P712 Ch2ab-KP_2_) and Sabin PV type 3 (Lot 457-III-Pfizer) were used.

Studies on virus culture conditions were carried out in 5-L glass bioreactors (Sartorius Stedim Biotech). Cell cultures were done in EMEM supplemented with bovine serum (BS) and 3 g L^-1^ micro-carriers (Cytodex 1; GE Healthcare) with the following settings: T of 37°C, pH of 7.2 and DO (dissolved oxygen) of 50% by headspace aeration. Glucose was added daily when the concentration was below 5 mM. Prior to virus culture the media was exchanged to M199. Virus cultures conditions were: T of 32.5 or 33.5°C, pH of 7.4, DO of 25% by headspace aeration. 

#### Downstream processing

Virus was harvested, clarified, concentrated, purified, first on size using size exclusion chromatography (SEC) and second by ion exchange chromatography (IEX), and finally inactivated as described previously [13]. 

#### Analytics

Cell counts were determined using a Nucleocounter (Chemometec). Glucose concentration was determined using a Bioprofile 100 plus (Nova Biomedical, MA). Cytopathic effects (CPE) were monitored microscopically. Virus was quantified by titer measurements (CCID_50_) [21] and by a modified D-antigen ELISA [22] for in-process samples.

### Clinical lots manufacturing

#### Cell and virus culture

Vero cells from a manufacturers working cell bank were used. Master and working seedlots were prepared from Sabin type 1 LSc 2ab KP_2_ (WHO/Beringwerke SO+1, 1976), type 2 P712 Ch2ab-KP_2_ (WHO/Behringwerke SO+1, 1976) and type 3 (Pfizer RSO1, SO+5, lot 457-III, 1963; supplied by Institute Mérieux to RIVM in 1991) [23]. Working seedlots were additionally tested for neurovirulence in monkeys (Bio Farma, Indonesia) [24] and analyzed with MAPREC (mutant analysis by PCR and restriction enzyme cleavage; NIBSC/HPA, UK) [25] and RCT40 (replicating properties 36°C- 40°C; AFSSAPS, France) [24] to assess genetic stability with respect to biosafety (Table 1).

**Table 1:** Biosafety and viral safety testing of Sabin PV master (MS) and working (WS) seedlots.

**Seedlot**	**Virus titer (Log_10_ CCID_50_ mL^-1^)**	**Monkey Neurovirulence^1^**	**MAPREC^2^**	**RCT40^1^**	**Extraneous agents/Viral safety^3^**
MS PV type 1	8.85	Not determined	Conform	Conform	Conform
MS PV type 2	7.52	Not determined	Conform	Conform	Conform
MS PV type 3	8.23	Not determined	Conform	Conform	Conform
WS PV type 1	8.90	Conform	Conform	Conform	Conform
WS PV type 2	7.55	Conform	Conform	Conform	Conform
WS PV type 3	8.45	Conform	Conform	Conform	Conform

^1^ Reproductive Capacity at 40°C Temperature (RCT40) and Monkey Neurovirulence: Tests and requirements according to WHO recommendations for OPV [24]. ^2^ Mutant Analysis by PCR and Restriction Enzyme Cleavage (MAPREC): Test and requirements according to new WHO recommendations for OPV [25]. ^3^ Test and requirements according to the European Pharmacopoeia [31,43].

Cell and virus culture was carried out in two 350-L (working volume) bioreactors. In short, thawed Vero cells were used to directly inoculate a 15-L fed-batch pre-culture (EMEM supplemented with BS and Cytodex 1 microcarriers) [26]. After trypsinization [26]; a 2^nd^ pre-culture using the recirculation culture method [27] was done to have sufficient cell to inoculate two 350-L bioreactors at 0.1 × 10^6^ cells mL^-1^. After medium exchange [28], virus culture was started (M199; Multiplicity of infection (MOI)=0.01; T=32.5°C).

#### Purification

Virus from the two 350-L (working volume) bioreactors was harvested via a sieve (mesh 75 µm) to obtain a virus harvest free of micro-carriers [13]. Clarification was done using normal flow filtration with a Millipore POD-holder containing C0HC depth filters followed by an Express SHC 0.45/0.22 µm combination filter (Millipore) [3,13] . Concentration was done by tangential flow filtration using 100 kDa filters [13]. Purification was done by size exclusion chromatography (Sepharose CL-6B (GE Healthcare) [11,29]; elution buffer 20mM phosphate buffer pH 7.0) and ion exchange chromatography (DEAE-Sephadex A50 (GE Healthcare) [11,30]; elution buffer 20mM phosphate buffer pH7.0). 

#### Inactivation

Purified virus was stabilized with concentrated M199 containing glycine (final conc. 5g L^-1^). Inactivation was done following the standard method: 0.025% formaldehyde incubation for 13 days at 37°C. An intermediate filtration (0.22 µm) was performed at day 6-8 [5]. The resulting monovalent bulk was stored at 2-8°C.

#### Preparation of trivalent vaccine

Monovalent bulks of PV type 1, 2 and 3 were mixed to a ratio of 60:96:192 D-antigen prior to sterile filtration. The sIPV final bulk was subsequently prepared by addition (via 0.22 µm filter) of the mixed trivalent bulk and dilution buffer containing phosphate, phenoxyethanol and formaldehyde. Aluminium hydroxide adjuvanted sIPV final bulk was prepared as described above with the inclusion of the addition of sterile Alhydrogel (Brenntag) (directly) to the final bulk. Final bulks were mixed for 10 minutes prior to setting the pH. The final lots were prepared by filling the final bulk in aliquots of 0.6-0.7g in 3 mL sterile siliconized vials using a Bausch & Ströbel ksf 1027 machine.

#### Analytics

During the process, sampling was done, as required for release of sIPV final lots. In Table 2 a list of the most relevant assays for release is given. A qualified QC department carried out these assays.

**Table 2:** Release requirements^1^ of plain and adjuvanted sIPV.

**Cell culture**		
Identity	Vero cells	
Mycoplasma	Absent	
Extraneous viruses	Absent	
**Virus harvest**		
Sterility	Absence of growth (Tryptic Soy Broth & Thioglycollate broth)	
Mycoplasma	Absent	
Extraneous viruses	Absent	
**Purified virus**		
Purity (ratio total protein and D-antigen)	≤0.1 µg DU^-1^	
Sterility	As above	
Identity	PV type 1, 2 OR 3	
Residual host cell proteins	Consistent clearance	
**Monovalent Bulk**		
Inactivation kinetics	PV titer below detection limit after 120h	
Formaldehyde	>2 mM	
Sterility	As above	
PV identity	Contains PV type 1, 2 OR 3	
Inactivation	Full absence of active PV after 10 and 13 days	
D-antigen content	Information for calculation	
**Trivalent bulk**		
Inactivation	Full absence of active PV in 1,500 calculated human doses	
Sterility	As above	
D-antigen content	Information for calculation	
**Final bulk**	**Plain**	**Adjuvanted**
pH	6.8-7.4	6.8-7.4
Phenoxyethanol	31-42 mM	31-42 mM
Formaldehyde ^2^	0.7-2.4 mM	0.7-1.3 mM
Sterility	As above	As above
D-antigen content	≥75% nominal value	≥75% nominal value
**Final lot**	**Plain**	**Adjuvanted**
Appearance	Bright red-orange fluid	Turbid red-orange fluid
PV identity	Contains type PV 1, 2 and 3	Contains type PV 1, 2 and 3
D-antigen content	≥75% nominal value	≥75% nominal value
Residual host cell DNA ^3^	≤100 pg shd^-1^	≤100 pg shd^-1^
Bovin Serum Albumin ^3^	≤50 ng shd^-1^	≤50 ng shd^-1^
Total protein	≤20 µg mL^-1^	≤20 µg mL^-1^
Endotoxin	≤10 IU mL^-1^	≤10 IU mL^-1^
Extractable volume	≥0.5 mL	≥0.5 mL
pH	6.8-7.4	6.8-7.4
Sterility	As above	As above
Free D-antigen	Not applicable	< 1%
Aluminium	Not applicable	0.26-0.36 mg mL^-1^
Abnormal toxicity (in mice and guinea pigs)	No illness	No illness

^1^ Most important release tests drafted for the production of the phase I clinical lots are given. It should be noted that the release criteria could change due to further product development. ^2^ Formaldehyde requirement is dependent on the amount of monovalent bulk used to prepare the final bulk. ^3^ Test is performed at an earlier stage in view of the lower detection limit.

Cell counts were performed using a hemocytometer. Cythopathic effects (CPE) were monitored microscopically. Virus was quantified by titer measurements (CCID_50_) and D-antigen ELISA [14] for release. SDS-PAGE was done using precast 4-20% gradient gel (Pierce) with Tris-HEPES buffer (Pierce). Vero host cell protein concentrations were determined using a Vero Cell HCP ELISA kit F500 according to the manufacturer’s instructions (Cygnus Technologies, NC). MAPREC and RCT40 were performed as described in WHO guidelines [24,25].

#### Preclinical studies - Rat immunogenicity

The rat immunogenicity was determined essentially as described previously [31,32]. In short, TOX rats (weighing 175-250g) that were screened for the absence of PV antibodies were injected intramuscularly with 0.5 mL of prepared vaccine dilutions (group size 10 per dilution). Blood samples were taken 21 days after injection. Collected sera (stored at -20°C until use) were analyzed for neutralizing antibodies. Serial dilutions (with M199) of heat inactivated sera (30 min 56°C) were prepared in a 96-wells plate and incubated with 50 µl (2 × 10^3^ TCID_50_ mL^-1^) PV type 1 Mahoney, PV type 2 MEF-1, or PV type 3 Saukett for 3h at 35-37°C in a CO_2_ incubator and subsequently stored overnight at 2-8°C. After addition of Vero cells (50 µL at 2 × 10^5^ cells mL^-1^) the 96-wells plates were incubated for 7 days at 35-37°C. Supernatants were discarded and cells were stained with a crystal violet solution containing 5% formaldehyde. Presence of full monolayers of Vero cells indicated a complete neutralization of the virus. The neutralization antibody score represents the highest dilution (log_2_; with a test maximum of 12) where complete neutralization was observed. For comparison of sIPV with conventional IPV, the international standard PU91-01 was diluted towards the conventional IPV dose (40/8/32 DU/shd).

#### Preclinical studies - Toxicity study in rabbits

A repeated dose and local tolerance toxicity study followed by a two week recovery period was carried out in rabbits (NOTOX, the Netherlands) according to EMA guidelines [33]. In short, New Zealand white rabbits (group size of 16, equally distributed among sexes) were treated by intramuscular injection with 0.5 mL vaccine or placebo (vaccine without D-antigen) at day 1, 15, 29, 43 and 57). Animals were necropsied at day 60 (n=10) or day 71 (n=6). The following observations and examinations were evaluated: clinical signs (daily), skin irritation (24 and 48 hours after each administration), body weight (weekly), food consumption (twice weekly), ophthalmoscopic examination (during pretest, end of treatment and end of recovery), rectal body temperature (during pretest, prior to each dosing and approximately 2 hours after dosing), clinical pathology (Pretest, Days 4, 57, 60 and 71), macroscopy at termination and organ weights and histopathology on a selection of tissues. 

### Statistical analysis

Two sided Student t-tests were performed with α=0.05. Numbers are given as means with standard deviations.

To determine the regression line slopes, no weighted regression was used. This was chosen based on the use of medians when concerning animal tests in contrast to the use of the means. In addition, the observed standard deviation from the median did not increase with increasing values. The significance (95% confidence) of the slopes was tested using an extra sum of squares F-test with the null hypothesis being a horizontal line (i.e. slope=0) (Graphad Prism 6 for Windows).

## Results

### Drafting product and process specifications

The specifications (Table 2) for the release and control of sIPV and aluminium hydroxide adjuvanted sIPV were drafted based on WHO [5] and EP monograph [31] for IPV manufacturing. Some product requirements, like formaldehyde content and pH, were based on the available IPV experience. Requirements related to the adjuvation with aluminium hydroxide were set after initial research [34]. The WHO OPV guidelines [24] were taken into account to assess the Sabin PV genetic stability with respect to biosafety, i.e. temperature sensitivity and revertants. 

### Process development prior to manufacturing of clinical lots

In view of the relatively short timelines in the polio eradication program it was chosen to prepare a sIPV with limited process development time prior to production of clinical lots. Process development therefore focused on Sabin strain specific adaptations like MOI and virus cultivation temperature and chromatography (discussed below). In addition, a disposable clarification unit was introduced to replace the Celite cake for depth-filtration. On all other aspects the production process was similar to conventional IPV manufacturing.

#### Selection of MOI and virus cultivation temperature

In OPV manufacturing, the virus cultivation temperature for Sabin PV is lower (at a maximum of 35°C [24]) than the temperature used for wild-type PV in conventional IPV manufacturing (36-37°C) [18,35]. This lower temperature is required to ensure the temperature sensitivity of the Sabin PV and minimize revertants to ensure a safe OPV. Although here an inactivated product has been developed, manufacturing itself should be biosafe and one of the prerequisites was to ensure the safety of the prepared virus harvest with respect to revertants of Sabin PV. 

The effects of virus culture temperature and multiplicity of infection (MOI) on the virus culture yields and culture time were assessed using PV type 2. No differences in virus yields were observed when the MOI was decreased from 0.1 to 0.01. Decreasing the temperature from 33.5°C to 32.5°C, had a negative effect on virus yields. Virus titers were 7.7 ± 0.1 (n=3) and 7.2 ± 0.1 (n=3) Log_10_ CCID_50_ mL^-1^ for cultures at respectively 33.5°C and 32.5°C. D-antigen (a measure for immunogenic virus) concentrations were 25 ± 3 (at 33.5°C) and 11 ± 5 DU mL^-1^ (at 32.5°C). Under all tested conditions virus culture was complete within 4 days, i.e. cytopathic effect (CPE) >90% and both virus titers and D-antigen (a measure for immunogenic virus) concentrations remained constant. Despite the lower yields at 32.5°C, this cultivation temperature was selected for preparing the virus seeds and clinical trial material. This choice was made to minimize the risk of PV revertants. Since no difference was observed when using a lower MOI, an MOI of 0.01 was used as it is preferred since smaller amounts of virus working seedlots will be needed.

#### Chromatography resin selection and buffer strength

Initial process development was done to confirm the use of resins and procedures available for wild-type PV. The present SEC resin and procedure were applicable for use with Sabin PV (data not shown). For IEX a choice between two validated resins needed to be made. Both DEAE Sephadex A-50 [30] and DEAE Sepharose Fast Flow [13] have been used for purification of PV. In both cases impurities are captured while Salk PV flows through. Initial studies using Sabin PV Type 1 showed some non-specific binding of the PV to DEAE Sepharose Fast Flow; this was confirmed for Sabin PV Type 2. The alternative resin DEAE Sephadex A50 allowed efficient separation of Sabin PV type 1, 2 and 3.

During the proof of principle study [23] in which OPV bulks were obtained to generate IPV, a precipitate was noticed during inactivation. Analysis showed that this precipitate was a phosphate based precipitate without product. The main source of the phosphate was traced back to the purification process where a 40mM phosphate buffer was used during chromatography (both SEC and IEX). Application of a weaker phosphate buffer (20mM) and the impact on product elution and inactivation was assessed. Product elution profiles in SEC and IEX using a 20mM phosphate buffer were comparable with those obtained after eluting with a 40mM phosphate buffer. Inactivation of virus eluted with 20mM phosphate was comparable while precipitates were absent.

### Manufacturing of clinical lots

#### Preparation of virus seed

Sabin PV strains closest to the Sabin original strains (PV T1: SO+1 Behringwerke 1976; PV T2: SO+1 Behringwerke 1976; and PV T3: RSO+1 Pfizer 1963) were used to produce new virus master seedlots at 10-L scale. The virus working seedlots were produced at 350-L scale [3]. These seeds were tested for neurovirulence using the monkey neurovirulence test [24]. However, due to limited global test capacity and to minimize costs it was chosen to only test the working seedlots. It was argued that the working seeds represent, on a worst case base, the quality of the master seed with respect to neurovirulence. Next to passing the neurovirulence test, the master and working seedlots also passed the tests for extraneous agents, RCT40 (reproductive capacity at 40°C temperature) and MAPREC (Mutant Analysis by PCR and Restriction Enzyme Cleavage ) (Table 1).

#### Upstream processing

**Figure 2:**
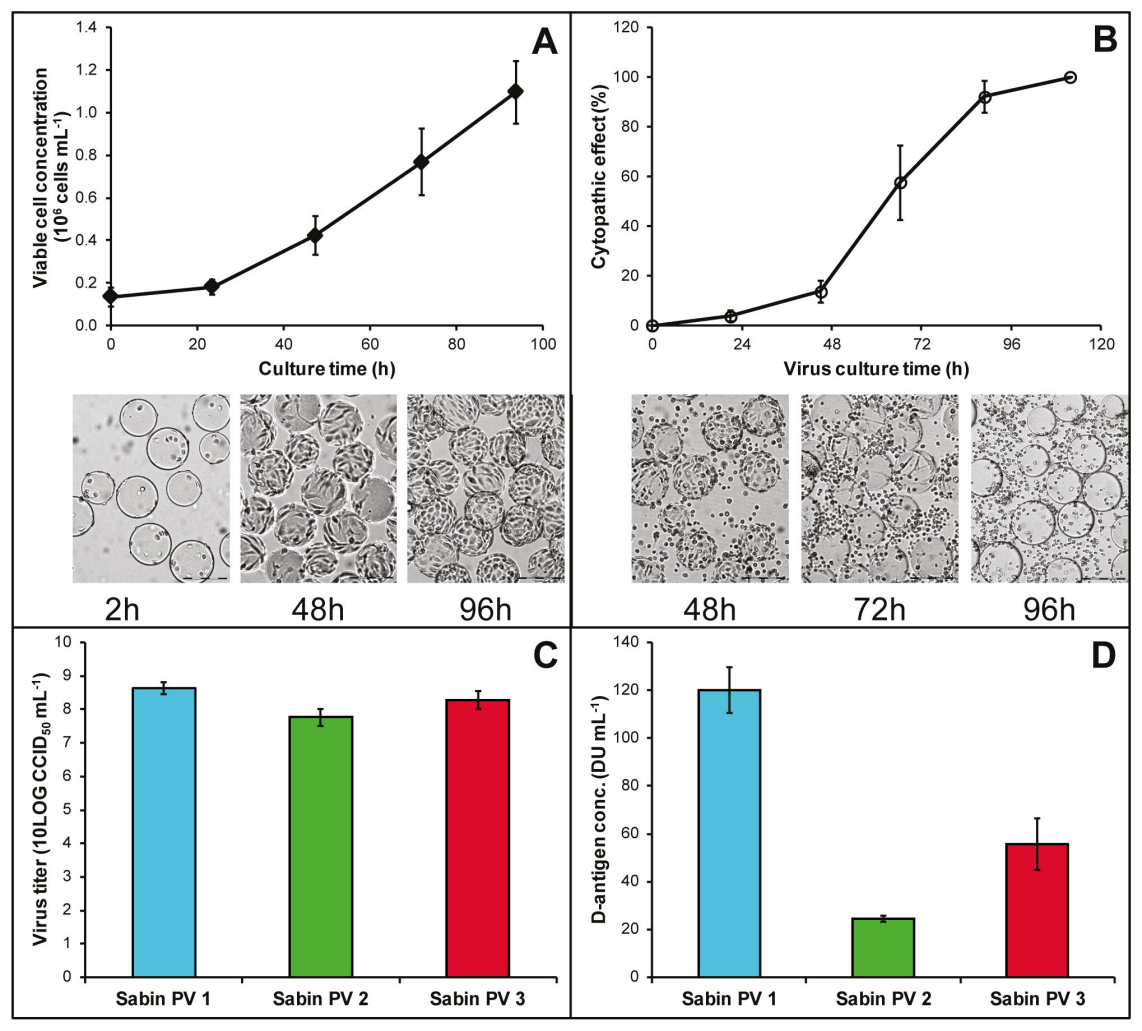
Cell and virus culture. Panel A shows the average Vero cell growth curve (n=12; error bars represent SD) in 350-L bioreactors. Photographs are light microscopy images (size bar 200 µm). Panel B shows the average (of the three subtypes) Vero cell death during virus culture determined microscopically (n=12; error bars represent SD). Photographs show corresponding images. Panel C shows average virus titers for Sabin PV type 1, 2 and 3 (n=4; error bars represent SD). Panel D shows average D-antigen concentrations after virus culture for Sabin PV type 1, 2 and 3 (n=4; error bars represent SD).

The preparation of sIPV was done in a physically separated production area in the established cGMP facilities for conventional IPV manufacturing. For each virus type two monovalent bulks were prepared. Vero cell culture was carried out in twin 350-L bioreactors [12]. In Figure 2A, the average growth curve of the 12 cultures (6 bulks in twin bioreactors) at 350-L scale is given. Cell culture was started at an inoculation density of 0.1 × 10^6^ cells mL^-1^. Cells grew adherent to micro-carriers (average growth rate 0.025 h^-1^) to reach 1.1 × 10^6^ cells mL^-1^ after 4 days when the micro-carriers were covered by a confluent layer of cells. At this point, the bovine serum containing medium was exchanged with serum free virus culture media. Subsequently, cells were infected with Sabin PV. Virus culture proceeded comparably and was independent of the virus type used for infection. Cell lysis was complete after 4-5 days as was determined based on the CPE observed microscopically (Figure 2B). Virus yields were based on virus titers (Figure 2C) and D-antigen (Figure 2D). While comparable virus titers were observed for the production of Sabin PV type 1 and 3, the yields for Sabin PV type 2 were significantly lower (2-tailed t-test; α=0.05 p=0.0043). D-antigen yields are not comparable between virus types as they are type (and antibody) specific [36]. From Figure 2C&D it was concluded that virus cultures were reproducible. The virus harvests were negative for revertants of Sabin PV as analyzed by RCT40 and MAPREC.

#### Downstream processing

Virus from the twin bioreactors was harvested and pooled prior to purification. Harvested virus was first clarified using normal flow filtration (NFF), which resulted in a decrease in fluid turbidity from 54 ± 6 NTU (Nephelometric Turbidity Unit) to 0.4 ± 0.4 NTU (n=5; determined mid-processing). The clarified virus was subsequently concentrated from approx. 700L to 1L using tangential flow filtration (TFF). Product recoveries, based on D-antigen units (DU), during the filtration steps were 90% ± 3% and 68% ± 11% for respectively NFF and TFF (Table 3).

**Table 3:** Product recovery during processing of two batches for each serotype.

Virus subtype	Harvest	Clarification	Concentration	SEC^2^	IEX^3^	Inactivation	Overall^1^
Sabin PV type 1	100%	86%	82%	62%	90%	84%	38%
	100%	89%	73%	67%	107%	85%	41%
Sabin PV type 2	100%	92%	77%	69%	20%	50%	<15%
	100%	96%	54%	51%	35%	64%	<15%
Sabin PV type 3	100%	91%	75%	75%	87%	36%	18%
	100%	88%	70%	83%	114%	72%	24%

Losses due to sampling were not considered for recovery calculations of individual unit operations. The overall product recovery includes losses due to sampling for in-process and release tests as well as sampling for research purposes.

^1^ The overall DSP yield was calculated by dividing the amount of D-antigen units of the monovalent bulk by the amount of D-antigen units from the harvest. ^2^ Size Exclusion Chromatography ^3^ Ion Exchange Chromatography

**Figure 3:**
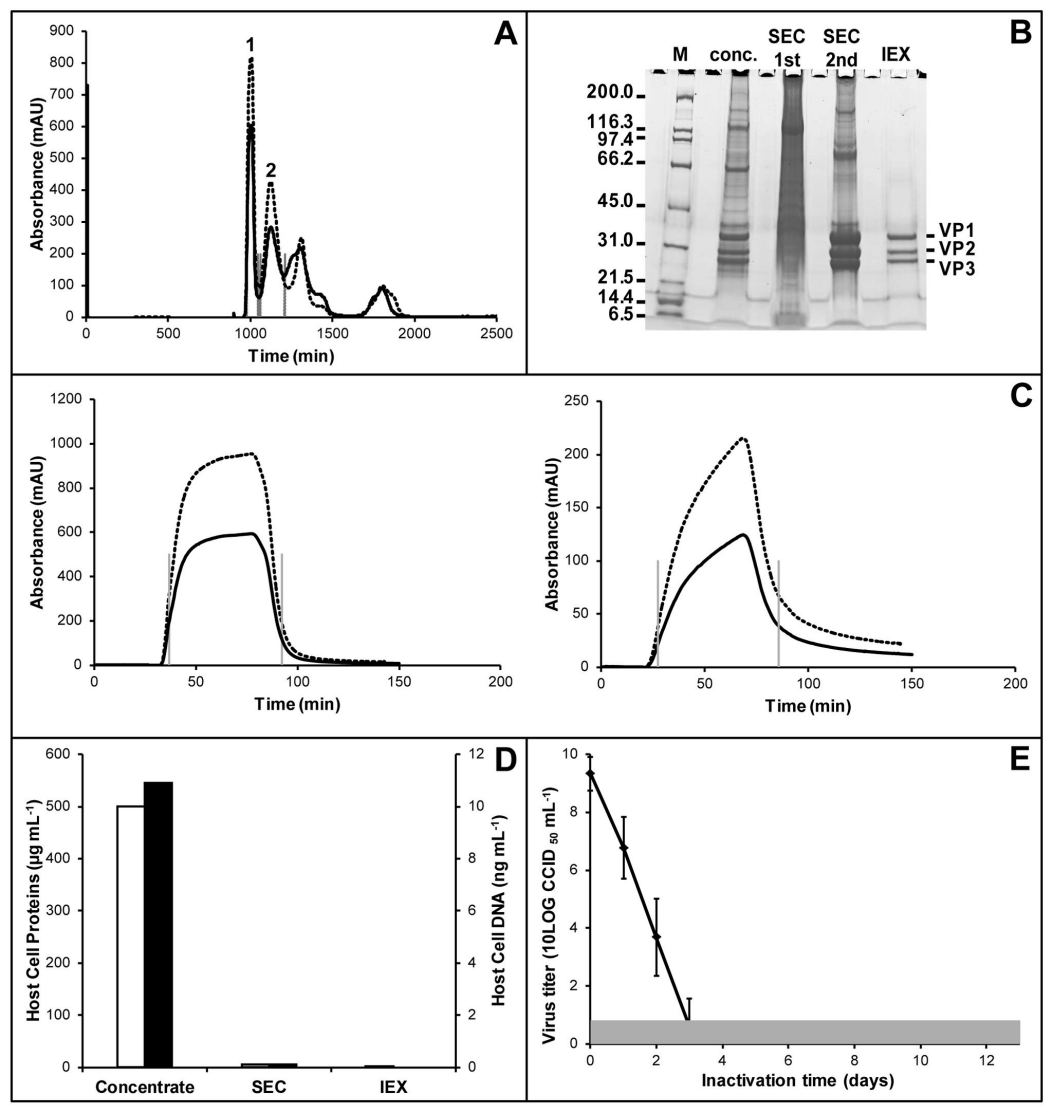
Purification of Sabin PV. Panel A depicts a SEC chromatogram of Sabin PV type 1. The 1^st^ peak contained mostly large cell components; the 2^nd^ peak contained the majority of PV, following peaks consist of smaller components. Panel B shows a SDS-PAGE (4-20% gel); lanes represent (from left to right) the marker, the concentrated product, followed by the 1^st^ and 2^nd^ fraction of SEC and finally the IEX purified PV. Panel C shows chromatograms of Sabin PV type 1 (left) and Sabin PV type 2 (right) IEX purification. Panel D shows host cell protein (open) and DNA (solid) impurities. Panel E depicts the inactivation of PV, the gray area indicates the lower detection limit. In chromatograms A and C, the dotted and solid lines represent absorbance at respectively 254nm and 280nm. Gray dotted lines indicate peak fractioning.

The concentrated product was purified using 2-step chromatography starting with SEC. In Figure 3A a typical SEC elution pattern for Sabin PV is given. The 1^st^ peak contains mostly large cell components. PV is found in the 2^nd^ peak as is illustrated by SDS-PAGE (Figure 3B) where the presence of the viral proteins is more pronounced when purification with SEC was done. An average D-antigen recovery of 68% ± 11% was found for SEC (Table 3). 

Subsequently the negatively charged molecules, like nucleic acids and proteins, were removed using IEX chromatography. PV should not interact with the matrix as was the case for Sabin PV type 1 and 3, where a plug flow was observed (Figure 3C). However, Sabin PV type 2 showed some interaction with the matrix as is apparent from the chromatogram (Figure 3C). The presence of the plug flow type chromatogram for Sabin PV type 1 and 3 allowed collection of the PV after IEX without major losses. Sabin PV type 1 and 3 were collected with 99.5% D-antigen recovery (Table 3). Relatively high losses (72%) were observed during IEX for Sabin PV type 2 (Table 3). The efficiency of the purification is illustrated by the SDS-PAGE in Figure 3B. After IEX the viral proteins are clearly purified from the other protein present after SEC. Removal of impurities was also shown for Vero host cell proteins and host cell DNA (Figure 3D). After IEX host cell protein concentrations were below 0.3 µg mL^-1^, corresponding to an over 1,000 times removal. Host cell DNA concentrations were below the detection limit of 78.13 pg mL^-1^ which is below the maximum level allowed in a single human dose (shd) (Table 2).

After IEX the Sabin PV was inactivated during a 13-day incubation period with formaldehyde. PV was inactivated rapidly, i.e. within 4 days, as shown in Figure 3E. After 6 to 8 days an intermediate filtration step was carried out to remove possible aggregates and ensure full inactivation. After inactivation a large variation in D-antigen recovery was observed, especially for Sabin PV type 3. Overall recoveries ranged from acceptable (in conventional IPV manufacturing on average approximately 40% for all three sub-types [13,37]), for Sabin PV type 1 (at 40%) to very low, with respect to future cost competitive processing, for PV type 2 (at Table 3). The obtained monovalent bulks met all release criteria and were stored at 4°C prior to mixing for formulation.

#### Formulation

Monovalent bulks were mixed to a trivalent bulk (Sabin PV type 1-2-3) in a ratio of 60-96-192 DU mL^-1^ prior to formulation to a final bulk. Different final bulks were prepared. Plain sIPV was prepared in different final concentrations of D-antigen to be able to test low (5-8-16 DU shd^-1^) middle (10-16-32 DU shd^-1^) and high (20-32-64 DU shd^-1^) dosages in (non-)clinical studies. Aluminium (Al(OH)_3_) adjuvanted vaccine was mixed at 2-fold lower D-antigen values being: low (2.5-4-8 DU shd^-1^), middle (5-8-16 DU shd^-1^) and high (10-16-32 DU shd^-1^). Vaccine was filled in vials as 0.5 mL per single human dose.

### Pre-clinical studies

**Figure 4:**
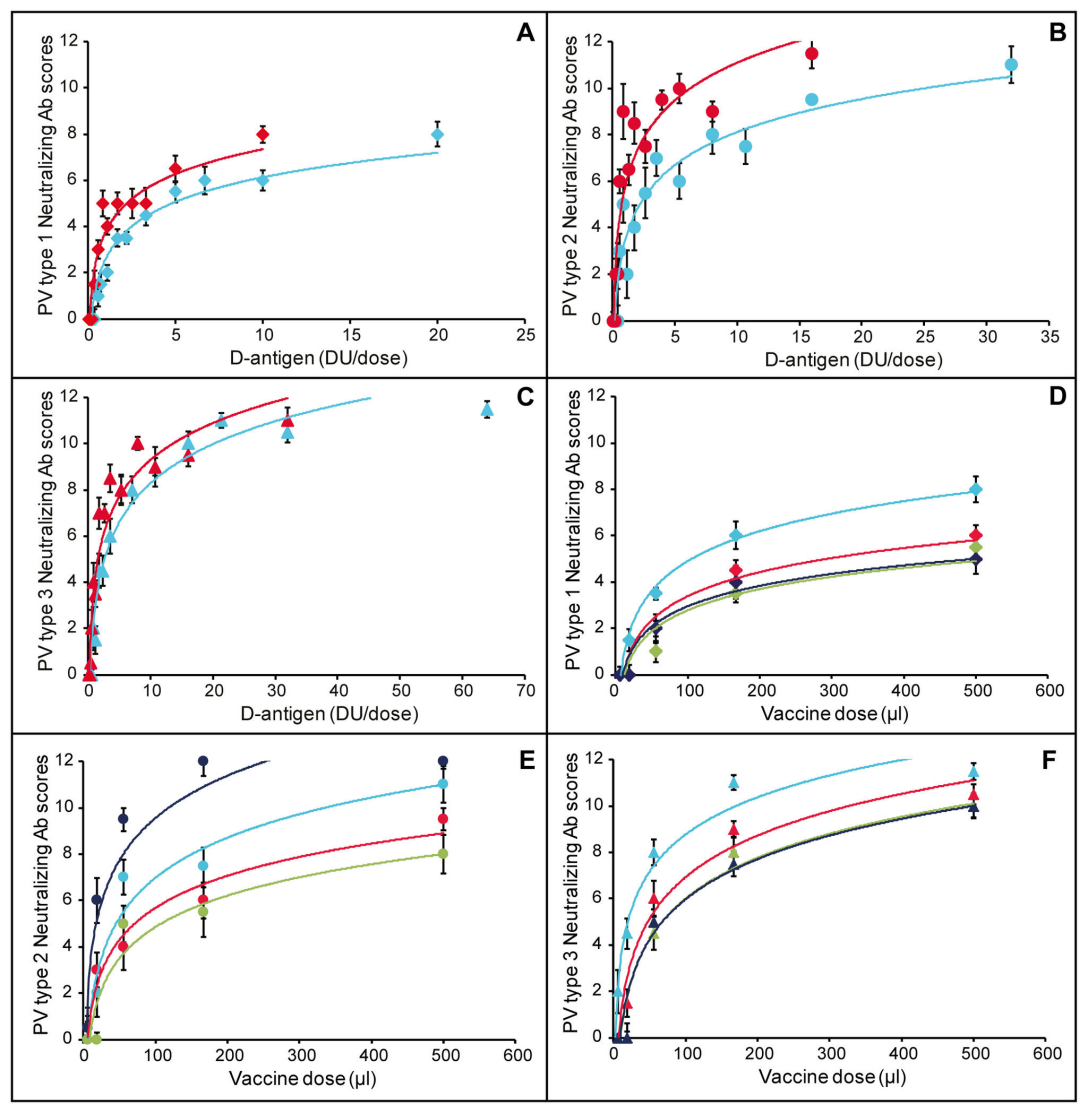
Rat immunogenicity (VNT against wild-type viruses). Panel A, B and C: VNT (log_2_ titer) to immunization with plain sIPV (blue) and adjuvanted sIPV (red) for PV type 1, 2 and 3 respectively; Panel D, E and F: VNT of plain sIPV 20/32/64 (light blue), 10/16/32 (red), 5/8/16 (green) and plain IPV 40/8/32 (dark blue) for PV type 1, 2 and 3 respectively. Error bars in panel A-F depict standard deviation of the median (n=10 rats).

The immunogenicities in terms of the capacity to induce virus neutralizing antibody titers (VNT) against the wild-type PVs (PV Type 1 Mahoney, PV Type 2 MEF-1 and PV Type 3 Saukett) of the six differently formulated vaccines were determined in rats [31,32]. High VNT against wild-type viruses were observed for all prepared formulations (i.e. 0.5 mL of high, middle and low DU). The maximum VNT for PV type 1 was lower than for PV type 2 and 3 (Figure 4A-C). For all types, the VNT increased with the dose and the addition of aluminium as adjuvant had a positive effect. For PV type 2 this effect was larger than for PV type 1 and PV type 3. Compared to conventional IPV, immunization of rats with sIPV resulted in comparable wild-type VNTs for PV type 1 and 3. Lower VNTs were found for PV type 2 when immunized with sIPV, however the levels of antibodies raised are very high (>8 log_2_) (Figure 4D-F). These data suggest that sIPV may be able to raise sufficient protective antibodies against all PV sub-types in humans, where the threshold for protection is 3 log_2_ [38] and thereby would be non-inferior to conventional IPV.

**Figure 5:**
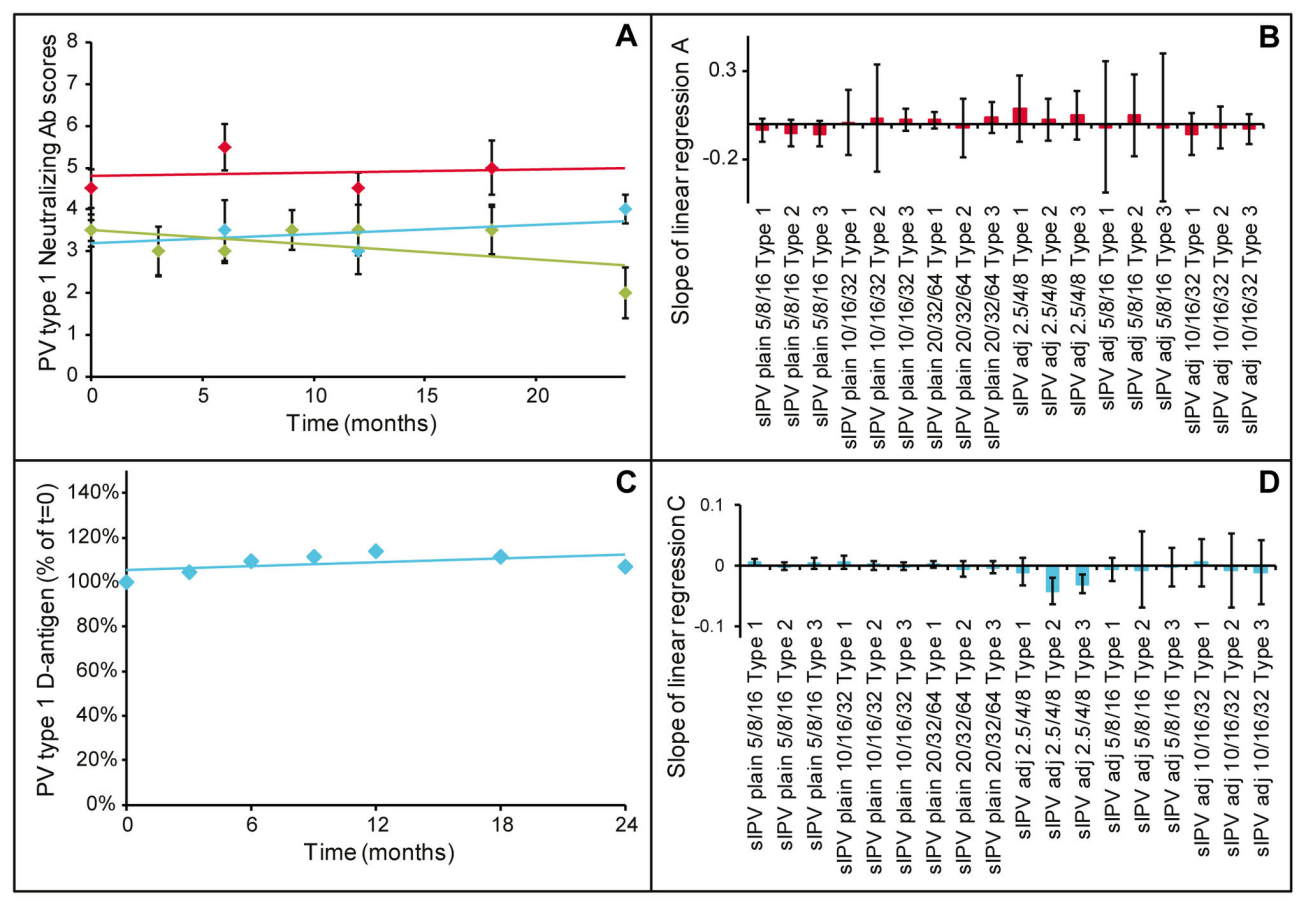
Stability of sIPV. Panel A: PV type 1 VNT of plain sIPV 20/32/64 (blue), 10/16/32 (red), 5/8/16 (green) in time, error bars depict standard deviation of the median (n=10 rats); Panel B: slopes of linear regression lines determined for stability based on rat immunogenicity as illustrated in panel A, error bars depict 95% confidence interval; Panel C: Stability of PV type 1 D-antigen of sIPV 20/32/64; Panel D: slopes of linear regression lines determined for stability based on D-antigen as illustrated in panel C, error bars depict 95% confidence interval.

Stability of the clinical lots over a period of 24 months was assessed based on immunogenicity in rats, D-antigen concentration, amount of free D-antigen (in case of adjuvanted vaccine) and more general parameters like sterility, appearance and pH. Vaccine stability with respect to immunogenicity in rats is illustrated in Figure 5A-D. Based on the regression line slopes (derived from Figure 5A) and their 95% confidence intervals (Figure 5B) it was concluded that all formulated clinical lots were stable with respect to immunogenicity in rats (null hypothesis slope=0; α=0.05; result p>0.05). 

In a similar way, the stability of the D-antigen content in the formulated clinical lots was reviewed. Slopes and their 95% confidence intervals of the regression lines (as illustrated in Figure 5C) were calculated. The measurement of D-antigen in aluminium adjuvanted formulations was difficult as the D-antigen needed to be desorbed from the aluminium prior to performing the D-antigen ELISA. This hurdle is illustrated by the larger confidence intervals found for the regression lines for D-antigen stability for the adjuvanted vaccines (Figure 5D). As a result no conclusions with respect to D-antigen stability could be drawn for the adjuvanted vaccine. Stability regarding the D-antigen content of the non-adjuvanted (plain) vaccine was good (null hypothesis slope =0; α=0.05; result p>0.05).

A repeated dose and local tolerance toxicity study in rabbits was conducted. Highest dose plain and adjuvanted sIPV were compared to a placebo and conventional licensed IPV. Some enlargement in local lymph nodes was found in all vaccine treated groups. Generally, minimal to mild inflammation was observed microscopically at the injection sites of all groups and could be attributed to the injection trauma. More intense inflammation was shown in the adjuvanted sIPV group, which was, in contrast to the other groups, not diminished after the two week recovery period. This was solely attributed to the apparent persistence of the adjuvant. A longer recovery period should have been chosen. The changes at the injection sites as well as the changes noted in the local lymph nodes are common findings in intramuscular vaccine studies meaning that the vaccines are safe to use in clinical trials.

## Discussion

The polio eradication program strives to a switch from OPV to sIPV and currently at least one dose of IPV is recommended [39]. In view of the relatively short timelines in the polio eradication program it was chosen to prepare a sIPV with limited process development time prior to production of clinical lots. Based on the existing large scale IPV manufacturing process development of sIPV was achieved. Main operating differences were related to the observed precipitate during inactivation, intrinsic virus properties resulting in adjustments in tests (i.e. aluminium desorption prior to D-antigen quantification) and limits required with respect to biosafety (i.e. virus culture temperature). The final product met quality criteria and could be released for testing in the clinical phase I/IIa studies in adults and infants to show safety and proof of principle.

Although a sIPV with required immunogenicity and purity could be produced, the purification yields with respect to Sabin PV type 2 were very low. These low levels will not result in a cost competitive IPV product. However, in light of the polio eradication program, and to pursue the fast implementation of worldwide sIPV manufacturing, sIPV production was continued despite the low type 2 yields. In this way it could be illustrated whether such a product would be comparable or better for polio vaccination compared to the conventional IPV. 

The next step in the project is to transfer the manufacturing knowledge to current vaccine manufacturers in low- and middle income countries to replace the OPV production with sIPV production [3]. The presented manufacturing process is being optimized in parallel with technology transfer. As product registration for market authorization at local authorities will require local clinical studies, necessary process optimizations for an economically feasible product can be implemented prior to this stage.

Worldwide efforts in the development of sIPV have recently resulted in market authorization for two vaccines containing sIPV in Japan [40]. Further, the Institute of Medical Biology, Chinese Academy of Medical Sciences (Kunming, China) [41] is currently performing clinical phase III studies [42]. This parallel development of sIPV allows a solid base for future IPV availability and minimization of risks with respect to biosafety during manufacturing.

## References

[1] Salk D, Salk J (1984) Vaccinology of poliomyelitis. Vaccine 2: 59-74. doi:10.1016/S0264-410X(98)90035-4. PubMed: 6099644. 6099644

[2] Aylward B, Tangermann R (2011) The global polio eradication initiative: Lessons learned and prospects for success. Vaccine 29, Supplement 4: D80-D85. doi:10.1016/j.vaccine.2011.10.005. PubMed: 22486981. 22486981

[3] Bakker WAM, Thomassen YE, van’t Oever AG, Westdijk J, van Oijen MGCT et al. (2011) Inactivated Polio Vaccine development for technology transfer using attenuated Sabin poliovirus strains to shift from Salk-IPV to Sabin-IPV. Vaccine 29: 7188-7196. doi:10.1016/j.vaccine.2011.05.079. PubMed: 21651934. 21651934

[4] Sabin AB, Boulger LR (1973) History of Sabin attenuated poliovirus oral live vaccine strains. J Biol Stand 1: 115-118. doi:10.1016/0092-1157(73)90048-6.

[5] WHO (2002) Recommendations for the production and control of poliomyelitis vaccine (inactivated) Technical Report Series No. 910 World Health Organization.

[6] WHO (2009) WHO global action plan to minimize poliovirus facility-associated risk after eradication of wild polioviruses and cessation of routine OPV use (Draft 2009). Available: www.polioeradication.org. Accessed 16 September 2013

[7] WHO (2004) Guidelines for the safe production and quality control of inactivated poliomyelitis vaccine manufactured from wild polioviruses Addendum, 2003, to the Recommendations for the Production and Quality Control of Poliomyelitis Vaccine (Inactivated) Technical Report Series No.926 World Health Organization.

[8] Venczel L, Landry S, Aylward B, Sutter R, Sabow A et al. (2009) Global post-eradication IPV supply and demand assessment: integrated findings commisioned by the Bill & Melinda Gates Foundation and prepared by Oliver Wyman Inc. Available: Available online at: www.polioeradication.org Accessed 16 September 2013

[9] Kew OM, Sutter RW, de Gourville EM, Dowdle WR, Pallansch MA (2005) Vaccine-derived polioviruses and the endgame strategy for global polio eradication. Annu Rev Microbiol 59: 587-635. doi:10.1146/annurev.micro.58.030603.123625. PubMed: 16153180. 16153180

[10] van Wezel AL (1967) Growth of cell-strains and primary cells on micro-carriers in homogeneous culture. Nature 216: 64-65. doi:10.1038/216064a0. PubMed: 4292963. 4292963

[11] van Wezel AL, van Herwaarden JA, van de Heuvel-de Rijk EW (1979) Large-scale concentration and purification of virus suspension from microcarrier culture for the preparation of inactivated virus vaccines. Dev Biol Stand 42: 65-69. PubMed: 223923. 223923

[12] Thomassen YE, van Sprang ENM, van der Pol LA, Bakker WAM (2010) Multivariate data analysis on historical IPV production data for better process understanding and future improvements. Biotechnol Bioeng 107: 96-104. doi:10.1002/bit.22788. PubMed: 20506395. 20506395

[13] Thomassen YE, van 't Oever AG, Vinke M, Spiekstra A, Wijffels RH et al. (2013) Scale-down of the inactivated polio vaccine production process. Biotechnol Bioeng 110: 1354-1365. doi:10.1002/bit.24798. PubMed: 23192424. 23192424

[14] Kersten G, Hazendonk T, Beuvery C (1999) Antigenic and immunogenic properties of inactivated polio vaccine made from Sabin strains. Vaccine 17: 2059-2066. doi:10.1016/S0264-410X(98)00409-5. PubMed: 10217607. 10217607

[15] Kreeftenberg H, Hamidi A (2007) Lessons learned in the transfer of Hib conjugate vaccine technology and the consequences for access to this vaccine in developing countries. In: WHO Meeting Report Intellectual Property Rights and Vaccines in Developing Countries, April 19–20, 2004 WHO Document WHO/IVB/0421.

[16] Kreeftenberg H, van der Velden T, Kersten G, van der Heuvel N, de Bruijn M (2006) Technology transfer of Sabin-IPV to new developing country markets. Biologicals 34: 155-158. doi:10.1016/j.biologicals.2006.02.011. PubMed: 16650773. 16650773

[17] van Noort RB (1992) The Children's Vaccine Initiative and vaccine supply: the role of the public sector. Vaccine 10: 909-910. doi:10.1016/0264-410X(92)90323-C. PubMed: 1471410. 1471410

[18] Duchene M, Peetermans J, D'Hondt E, Harford N, Fabry L et al. (1990) Production of poliovirus vaccines: past, present, and future. Viral Immunol 3: 243-272. doi:10.1089/vim.1990.3.243. PubMed: 2076176. 2076176

[19] Verdijk P (2012) Safety and Immunogenicity of a New Inactivated Polio Vaccine in Healthy Adults. In: Clinicaltrials.gov [internet]: Bethesda (MD): National Library of Medicine (US) 2000-[cited 2013 May 30]. Available: http://clinicaltrials.gov/show/NCT01708720 NLM Identified: NCT01708720.

[20] Verdijk P, Rots NY, Van Oijen MGCT, Oberste MS, Boog CJ et al. (2013) Safety and Immunogenicity of inactivated poliovirus vaccine based on Sabin strains with and without aluminum hydroxide: a phase I trial in healthy adults. Vaccine, 31: 5531–6. doi:10.1016/j.vaccine.2013.09.021. PubMed: 24063976. 24063976

[21] Thomassen YE, van der Welle JE, van Eikenhorst G, van der Pol LA, Bakker WAM (2012) Transfer of an adherent Vero cell culture method between two different rocking motion type bioreactors with respect to cell growth and metabolic rates. Process Biochem 47: 288-296. doi:10.1016/j.procbio.2011.11.006.

[22] ten Have R, Thomassen YE, Hamzink MRJ, Bakker WAM, Nijst OEM et al. (2012) Development of a fast ELISA for quantifying polio D-antigen in in-process samples. Biologicals 40: 84-87. doi:10.1016/j.biologicals.2011.11.004. PubMed: 22154015. 22154015

[23] Bakker WAM, Thomassen YE, ’t Oever AG, Westdijk J, Oijen MGCT et al. (2012) Development of Inactivated Polio Vaccine (IPV) Derived from Attenuated Sabin Strains. Proceedings of the 21st Annual Meeting of the European Society for Animal Cell Technology (ESACT), Dublin, Ireland, June. pp. 7-10, (2009)Jenkins N, Barron N, Alves P, editors. Springer Netherlands . pp. 667-670

[24] Expert Committee on Biological Standardization (2002) Fifthied report. Recommendations for the production and control of poliomyelitis vaccine (oral). Technical Report Series No.904 World Health Organization. pp. 31-93.

[25] WHO (2012) Recommendations to assure the quality, safety and efficacy of live attenuated poliomyelitis vaccine (oral). Replacement of TRS 904, annex 1 and addendum TRS 910, annex 1.

[26] van Wezel AL (1985) Monolayer growth systems: homogeneous unit processes. In: Spier RE Griffith JB Animal Cell Biotechnology. London: Academic Press pp. 265-282.

[27] van Hemert P, Kilburn DG, van Wezel AL (1969) Homogeneous cultivation of animal cells for the production of virus and virus products. Biotechnol Bioeng 11: 875-885. doi:10.1002/bit.260110513. PubMed: 5361174. 5361174

[28] van Wezel AL (1973) Microcarrier cultures of animal cells. Tissue culture, methods and applications VII Cell propagations on culture supports. New York and London: Academic Press pp. 372-376.

[29] van Wezel AL, van Steenis G, Hannik CA, Cohen H (1978) New approach to the production of concentrated and purified inactivated polio and rabies tissue culture vaccines. Dev Biol Stand 41: 159-168. PubMed: 223908. 223908

[30] van Wezel AL (1971) New trends in the preparation of cell substrates for the production of virus vaccines. Prog Immunobiol Stand 5: 187-192. PubMed: 4349819. 4349819

[31] European Pharmacopoeia 7.0 Monograph 0214 (2010). Poliomeylitis Vaccine (Inactivated) Strasbourg, France.

[32] van Steenis G, van Wezel AL, Sekhuis VM (1981) Potency testing of killed polio vaccine in rats. Dev Biol Stand 47: 119-128. PubMed: 6262142. 6262142

[33] European Agency for the Evaluation of Medicinal Products/Committee for Proprietary Medicinal Products (EMEA/CPMP) (1998). Note for guidance on preclinical pharmacological and toxicological testing of vaccines (CPMP/SWP/465/95, June 1998).

[34] Westdijk J, Koedam P, Barro M, Steil BP, Collin N et al. (2013) Antigen sparing with adjuvanted inactivated polio vaccine based on Sabin strains. Vaccine 31: 1298-1304. doi:10.1016/j.vaccine.2012.12.076. PubMed: 23313617. 23313617 PMC3570672

[35] Bakker WAM, Thomassen YE, van der Pol LA (2010) Scale-down approach for animal-free polio vaccine production. In: Noll T Cells and Culture; Proceedings of the 20th ESACT Meeting, Dresden, Germany: Springer Netherlands. pp. 541-550

[36] Westdijk J, Brugmans D, Martin J, Oever AV, Bakker WA et al. (2011) Characterization and standardization of Sabin based inactivated polio vaccine: Proposal for a new antigen unit for inactivated polio vaccines. Vaccine 29: 3390-3397. doi:10.1016/j.vaccine.2011.02.085. PubMed: 21397718. 21397718

[37] van Wezel AL, van SG, van der MP, Osterhaus AD (1984) Inactivated poliovirus vaccine: current production methods and new developments. Rev Infect Dis 6 Suppl 2: S335-S340. doi:10.1093/clinids/6.Supplement_2.S335. PubMed: 6429814. 6429814

[38] Vidor E, Plotkin SA (2013) Poliovirus vaccine-inactivated. In: Plotkin SA Orenstein WA Offit PA Vaccines. 6th ed. Elsevier.

[39] WHO (2013) Meeting of the Strategic Advisory Group of Experts on Immunization, November 2012 - conclusions and recommendations. Wkly Epidemiol Rec 88: 1-16. 23311010

[40] Tanimoto T, Murashige N, Hosoda M, Kusumi E, Ono S et al. (2012) Vaccination for whom? Time to reinvigorate Japanese vaccine policy. Lancet 380: 1647. doi:10.1016/S0140-6736(12)61949-7. 23141615

[41] Liao G, Li R, Li C, Sun M, Li Y et al. (2012) Safety and Immunogenicity of Inactivated Poliovirus Vaccine Made From Sabin Strains: A Phase II, Randomized, Positive-Controlled Trial. J Infect Dis 205: 237-243. doi:10.1093/infdis/jir723. PubMed: 22158682. 22158682

[42] Liao G, Li Y, Li C (2012) The Clinical Trial Protocol for the Inactivated Poliomyelitis Vaccine Made From Sabin Strains(Sabin IPV). Clinicaltrials.gov [internet]: Bethesda (MD) : National Library of Medicine ( US) 2000-[cited 2013 May 30]. Available: http://clinicaltrials.gov/show/NCT01510366 NLM Identified: NCT01510366.

[43] European Pharmacopoeia 7.0 (2011). Monograph 20616. Tests for extraneous agents in viral vaccines for human use.

